# Perceived health inequalities: are the UK and US public aware of occupation-related health inequality, and do they wish to see it reduced?

**DOI:** 10.1186/s12889-023-17120-6

**Published:** 2023-11-24

**Authors:** Emma K. Bridger, Angela Tufte-Hewett, David A. Comerford

**Affiliations:** 1 School of Psychology and Vision Sciences, University of Leicester, Leicester, England; 2 Department of Psychology, Birmingham City University, Birmingham, England; 3 Behavioural Science Centre, University of Stirling, Stirling, Scotland

**Keywords:** Occupational Health Inequality, Public awareness, Life Expectancy, Lay perceptions, Inequality aversion

## Abstract

**Background:**

One underexamined factor in the study of lay views of socioeconomic health inequalities is occupation-related health. Examining health by occupational social class has a long history in the UK but has been comparatively overlooked in US public health literatures, where the relationship between health and work has attended more to hazard exposure.

**Methods:**

Representative samples of the UK and US indicated the perceived and ideal lifespan of people working in “higher managerial/professional” and “routine” occupations. We examine perceptions of inequality and desires for equality across occupation groups as a function of country and key socio-demographic variables.

**Results:**

67.8% of UK and 53.7% of US participants identified that professionals live longer than routine workers. Multivariate models indicated that US participants were markedly less likely to be aware of occupation-related inequalities after controlling for age, gender, and education. Awareness was negatively related to age (in the US) and recent voting behaviours (both samples). Desiring equal life expectancy was less likely in the US sample, and less likely across both samples among older participants and those with lower levels of education.

**Conclusion:**

Employing a novel approach to measuring perceived and ideal life expectancy inequality, this is the first study to examine perceptions of lifespan inequality by occupational groups. It reports widespread understanding of the occupation-related gradient in lifespan and a desire that these inequalities be eliminated in the UK, but considerably less awareness and desire for equality in the US. Greater tolerance for social status inequalities in the US than other similar countries appear to also extend to differences in life expectancy.

**Supplementary Information:**

The online version contains supplementary material available at 10.1186/s12889-023-17120-6.

There has been substantial research activity into describing, explaining, and mitigating the fact that health outcomes are unequal across groups defined by socioeconomic factors [1, 2]. In line with the argument that successful reduction in health inequalities can only be achieved if there is public will to do so [3] and that public voices should be incorporated in public health research seeking to effect change, [4] there has been increased focus on understanding lay views of health inequalities [5] and trying to get “the public on board” with broader upstream policies that tackle the wider determinants of health [6–9]. Advocates of this approach appear to implicitly assume that the public already share their view that health inequalities should be reduced or even eliminated [10]. However, one recent public report focusing on this issue claims that the scale of health inequalities is a surprise to many people in the UK, and that although many report concern, health inequality appears to be ranked as less serious compared to other forms of social inequality [11].

A well-reported finding in psychological research on economic inequality is that preferences and attitudes towards redistribution policies are driven by people’s perceptions of inequality rather than actual or objective levels of inequality [12–15]. For instance, across a variety of multi-national survey samples, Gimpelson and Treisman [16] observe little correspondence between levels of actual and perceived economic inequality, and that the latter is more strongly associated with desires to redistribute wealth than actual levels of inequality. When it comes to public attitudes therefore, subjective perceptions of inequality matter more than objective inequality.

Also apparent from literatures on views of health inequality, is that the way in which health variation is framed influences people’s responses and particularly their aversion to it. Cases in which health inequality is presented with respect to another aggregating factor or domain (e.g. income-related health inequality) [17] [18], tend to elicit greater aversion to inequality than when health inequality is presented alone (without an aggregating factor, e.g., some people are more likely than others to live a long and healthy life) [17, 18]. This is particularly the case for socioeconomic framings of health inequality, such as income or wealth, relative to neutral or no framings [18, 19]. One comparatively underexamined socioeconomic factor in the study of lay perceptions of health inequality, however, is occupation. Whilst occupation is intimately related to other markers of socioeconomic status (SES) such as income and education, it has been argued that occupational prestige more directly measures social status or standing of the job holder [20], or at least is often perceived to do so [21], as well as workplace characteristics that may impact health such as hazard exposure [22] and power differentials [23]. Accordingly, measures of occupational class have independent links to a range of health outcomes, even after adjustment for education and income [24], and are likely to be capturing a different set of socioeconomic conditions to other socioeconomic measures [25, 26].

In the United Kingdom (UK) measures of social status and “occupational social class”^1^ have historically been closely intertwined. From 1931 to 2001, official statisticians in England and Wales employed the Registrar General’s Social Classes (RGSC) classification, which classified occupations into one of six classes, said to be based on ‘general standing in the community’. [2, 21] The scheme is considered hierarchical and an implicit measure of prestige, yet unlike other classifications [27] it was not derived from any clear definition or theory of social stratification making it difficult to articulate precisely how it relates to health. Despite being closer to ‘common sense’ views than theoretically-informed models [21], the RGSC was repeatedly employed to describe robust health inequalities in the UK, including in foundational work such as the Whitehall studies [28, 29]. From 2001 to 2011, the RGSC was replaced with the National Statistics Socio-economic Classification (NS-SEC), a seven-level schema derived from Goldthorpe’s Schema [30]. The scheme’s development reflects both the changing nature of work and makes explicit that whilst it represents theoretically meaningful elements of employment, it cannot represent all sources of socioeconomic inequality [21]. Key to the approach is the differentiation of core elements of employment relations such as supervision and control and the specificity of worker skills [30] Its theoretical grounding and relative simplicity means the NS-SEC has been used across multiple statistical domains in the UK, including health, until it was further rebased on the Standard Occupational Classification 2020 for the 2021 UK census [31].

In contrast, occupation has often been comparatively overlooked as a marker of socioeconomic status in the United States (US) [32–34]. Whilst occupation and social status have a long interlocking history in the UK, this is less the case in the US where social epidemiology arose from structural-functionalist perspectives that emphasised the fit of talents to society’s needs for occupational specialisms [2]. Occupational mobility was greater in the US than the UK from the nineteenth century at least until the 1940s [35] and this may extend to more recent history depending on how mobility is measured [36, 37] or conceptualised [38]. Accordingly, US public opinion is generally consistent with beliefs in high rates of mobility [39] regardless of whether high levels of occupational mobility truly continue to exist. This, combined with greater familiarity with income inequality, may contribute to greater relative emphasis on education and income, at the expense of occupation, as markers of status. In the context of health equity, observers have put forward several reasons for why work and occupation has been rather less explored in the US including the complexity of defining work and its relationships to health [32]. A particularly potent factor, is likely to be the partitioning of work-related health inequity into a distinct epidemiological community that focuses primarily on occupational health inequalities as a function of hazard exposure, with much less emphasis on the role of power and status [23, 32]. A recent meta-research report on leading international health inequalities research, also noted a greater focus on health disparities in the form of ethnic or racial gaps in health outcomes in the US relative to European studies [40]. Reduced focus on occupation is also compounded by limited data linking work and health outcomes in the US [32]. Accordingly, although links between occupation and health have been reported in the US [41, 42], these are much less frequently encountered than in Europe despite repeated calls for greater inclusion of the study of occupation and work in population and public health [32, 43, 44].

Public health focus on the role of occupational differences in health and longevity thus differs markedly in the UK and USA. If this difference in perspective extends to the broader public, then it may be expected that awareness of health inequalities as a function of occupational group will be greater in the UK than US. The objectives of the current research were to assess whether this is the case in representative samples of the UK and US public. To address this, we follow a paper that inferred respondents’ perceptions of and preferences for income inequality. It compared reports of ideal earnings for CEOs and “unskilled” workers and found that perceived pay inequality is less than actual pay inequality, but higher than ideal pay inequality [45]. We ask a panel of respondents who are representative of the UK and US populations to estimate the typical lifespan of people working as “higher managers/professionals” and in “routine” occupations. These labels correspond with two categories from the NS-Sect [46]. Accordingly, there exists administrative data in the UK on age at death from 2007 to 2011 for these groups, which show life expectancy advantages for higher managers/professionals relative to those in routine occupations ( [47]; see Table Table 1). We can therefore compare health inequality as perceived by our UK sample against an objective baseline.

A further advantage of this approach is it allows us to also ask the same respondents what the lifespan of these two groups would ideally be, in order to indirectly assess views on occupational health inequality. Note that while some studies have looked to answer related questions using choice experiments (e.g., [48–50]), this is not appropriate to the current research questions because choice experiments measure trade-offs whereas we seek to measure unconditional desirances (i.e., instead of asking people to choose an outcome, we elicit which outcome a population would rather receive, [51]). Although care is needed not to interpret these responses as the same as preferences for policies that reduce health inequalities [9], we nonetheless see value in capturing these unconditional desires because they are directly comparable with actual and perceived levels of inequality, and therefore provide an indication of whether desires overlap with actual or perceived inequality.

In summary, the key objectives are to report for the first time: (i) perceptions of life expectancy differences by occupation in representative samples of the UK and US public, (ii) how accurate the UK-public is in its perception of occupational social class health inequalities, (iii) how estimated occupational health inequality compares to ideal health (in)equality according to the UK and US population, (iv) which level of occupational health (in)equality that the UK and US population desire to see and (v) which socio-demographic and person-level factors are associated with the awareness of this kind of inequality as well as the desire to see lifespan equality.

## Methods

### Design and participants

YouGov Plc GB/US collected data from the two countries using their online panel, which includes over 7,000,000 persons who have previously consented to take part in surveys. YouGov use sampling quotas whilst the survey is in field, applying targets for a range of demographic variables so that the sample collected is representative of the national population. YouGov do not report response rates but ensure final samples remain representative by applying individual weights using these targets. These targets are derived from official, publicly available sources such as the census and actual election results. For the UK sample, targets and weights were based on age, gender, education level, political attention, social grade, past vote and region. For the US sample, targets and weights were based on age, gender, race and education. Although it is not possible to specify the initial response rate from this sampling procedure, the application of these weights to all analyses offsets some concerns about selection bias in these samples.

Survey responses were collected from UK panellists from 9th − 10th December 2020 and from US panellists from 11th -16th December 2020. Initial sample sizes were 1,741 and 1,301 for the UK and US, respectively. Final samples were derived by limiting inclusion to only those respondents whose responses for all four of the key life expectancy questions were within two standard deviations of the mean response for that question and sample. A greater proportion of the US sample (20.1%) did not meet these criteria compared to the UK sample (7.5%). YouGov calculated post-stratification weights for the final samples, and these were applied prior to conducting all descriptive and inferential analyses. The final samples were 1,599 UK adults and 1,039 US adults, with an age range of 18–90 (mean age = 48.17).

### Main measures

Due to its complexity, it was not feasible to provide participants with a full explanation of the NS-SEC. Instead, they were initially provided with the following context: “*In this study, we would like to learn about your perceptions of people in different occupational groups. The government uses a classification scheme to group people according to the kinds of job they have*.” To help participants understand the specific categories from this scheme, two occupations were selected from each category on the basis that these would be easy to conceptualise for respondents in either country and would not be strongly associated with adverse health outcomes or hazardous working conditions (e.g., coal miners). Specifically, participants were told: “*One group within this scheme is people with higher managerial and professional jobs, which includes engineers and veterinarians. Another group in this scheme are people with routine jobs, which includes construction workers and parking compliance officers*.” After this, respondents were asked: “*In 2007–2011, what do you think the typical lifespan (in years from birth) of people in higher managerial and professional jobs was?*” and “*In 2007–2011, what do you think the typical lifespan (in years from birth) of people in routine jobs was?*” Next, participants were asked “*What do you think the typical lifespan (in years from birth) of people in higher managerial and professional jobs should be?*” and “*What do you think the typical lifespan (in years from birth) of people in routine jobs should be?*“ Only numerical responses could be provided. Participants were always asked about higher managerial and professional jobs first and were always asked to give estimates prior to their ideal judgments.

### Socio-demographic measures

For the principal cross-country comparisons, we employed those variables that were available and harmonizable across both countries: participant gender, age, highest level of education and household income. Respondent age was categorised into five comparably-sized groups of 18–31 (19.1%), 32–45 (21.1%), 46–58 (21.3%), 59–67 (19.4%), 68+ (19%). UK data on highest level of education comprised 18 different options which were recoded to three levels: no formal qualifications or level 2–3 or equivalent (youth training certificate, trade apprenticeship, clerical and commercial, City & Guilds, City & Guilds advanced, ONC, CSE grade 1, CSE grades 2–5, Scottish Ordinary, A level/Higher, Scottish Higher), University degree or equivalent (Nursing qualification, Teaching qualification, University diploma, University degree) or post-graduate/higher (University higher degree, other technical, professional or higher qualification). The six levels provided for US education were recoded into three comparative levels of education: no formal education or high school, high school graduate, college level (some college, 2-year college, 4-years college) and post-graduate. UK annual household income was provided in £5,000 increments, however, US household income indicated only whether income was <$40,000, $40,000 to $80,000 or >$80,000. Harmonisation was achieved by calculating sterling equivalent categories for the three US levels based on exchange rate in February 2021 (<£30,000; £30,000-£60,000; >£60,000) and re-coding UK data accordingly.

In separate country-specific analyses we sought to further examine the role of additional variables unique to each panel. Accordingly, these analyses cannot be compared across countries but do permit further examination of subsequent socio-demographic variables. Following findings elsewhere showing that political orientation and ideology predicts perceptions of inequality [52] including views on lifespan inequalities between the rich and the poor, [53] we opted to include variables relating to political behaviours. In the YouGov UK panel this included measures of political attention and voting behaviour in the 2019 General Election. Political attention was a self-report measure (“How much attention do you generally pay to politics?” on a scale from 0 to 10), which was recoded to high (7–10), medium (5–6) and low (0–4), but otherwise was left continuous. Respondents indicated whether they voted Conservative, Labour or Other (Liberal Democrat, Scottish National Party, Plaid Cymru, Brexit Party, Green, other, Don’t Know) or did not vote in the General Election in December 2019.

In the US panel this comprised voting behaviour in the 2020 Presidential election, either for Joe Biden, Donald Trump, Other candidates (Jo Jorgensen, Howie Hawkins or Other) or Did not vote for President. YouGov UK panel data also included a measure of household Social Grade [54], developed by the National Readership Survey as a hierarchical scale often used in market research that closely resembles other occupational classes such as the NS-Sect [21]. This measure is based mainly but not only on occupation and employment status of Chief Income Earner and was categorised into high (A: higher managerial, administrative and professional, B: Intermediate managerial, administrative and professional), medium (C1: Supervisory, clerical and junior managerial, administrative and professional, C2: skilled manual workers) and low social grade (D: semi-skilled and unskilled manual workers, E: state pensioners, casual and lowest grade workers, unemployed with state benefits). As the best available measure on participants’ own occupation, we included this to determine whether this influenced participants’ views on lifespan differences between professionals and routine workers. Finally, given the emphasis on health inequity between different racial categories in the US, [40] we included these data for the US panel analyses. US respondents indicated which racial or ethnic group best describes them: White, Black, Hispanic or other persons of colour (Asian, Native American, Two or more races, Other, or Middle Eastern). No data on race or ethnicity were available for the UK sample.

### Analysis

To quantify estimated and desired levels of inequality, we calculated ratios such that each respondent’s estimate for professionals was divided by their estimate for those in routine occupations (as in [45]). Ratios > 1 therefore indicate greater life expectancy for professionals than routine workers, ratios of 1 indicate the same for both groups, and ratios < 1 indicate greater life expectancy for routine workers than professionals. Ratios failed to meet normal distribution assumptions, and therefore related samples Wilcoxon Signed-Rank Tests were used to compare estimated and ideal life expectancy ratios.

Respondents were coded as being aware of occupational life expectancy inequality if they provided life expectancy estimates that favoured professionals relative to routine workers (ratios > 1). Respondents were coded as desiring equal life expectancy between occupational groups if they provided life expectancy desirances that were equal for the two occupational groups (ratios = 1). We sought to determine which variables were associated with each of these outcomes firstly in a cross-country analysis with harmonizable variables (and country) and secondly in separate country-specific analyses. In the UK sample only, we also examined which variables were associated with being within +/- 2 years of the correct socioeconomic gradient in life expectancy. Analyses comprised multivariate logistic regressions with simultaneously entered socio-demographic predictors and were weighted using YouGov post-stratification weights (see Supplemental materials S1 and S3 for comparable analyses without weights). All predictors were modelled as categorical, and contrasts are reported relative to a single reference category. The significance level was set at 0.05. Adjusted odds ratios and 95% confidence intervals (95% CI) for all variables are reported. Generalized linear models were tested using R in RStudio and corresponding forest plots were created using the *forestmodel* package ( [55]; see data availability statement for access to data and code). A substantial proportion of both samples were missing data on household income (UK, n = 462, 28.9%; US, n = 153, 14.7%). Occasional data on socio-demographic variables other than household income were also missing in the UK sample, specifically on education (n = 64) and general election vote (n = 1). Missing data were imputed using a Random Forest estimation trained on observed values to predict missing values [56] that has been shown to have lower imputation error than other approaches [57]. For sensitivity purposes, supplementary materials (Figs S1-S4) include comparable analyses without statistical weights as well as on raw data excluding household income.

## Results

### Estimated and ideal life expectancy differences for professionals and routine workers

**Table 1:** Mean age in years (standard deviations in parentheses) reported by respondents from the UK (*N* = 1,599) and US (*N* = 1,039) for the two NS-SEC categories

	Actual	Estimated	Ideal
**UK Professional**	86.1	78.25 (7.06)	81.64 (7.83)
**UK Routine**	81.4	75.11 (6.74)	80.78 (7.97)
**UK Average Estimated Lifespan**		76.68 (5.88)	
**US Professional**	n/a	71.93 (14.69)	75.99 (16.88)
**US Routine**	n/a	70.32 (14.70)	75.36 (17.44)
**US Average Estimated Lifespan**		71.13 (13.86)	

Table 1 presents mean age in years reported by respondents and, for UK respondents, the actual average age of death for professional and routine workers between 2007 and 2011. UK respondents underestimated age of death for professionals (by about 8 years) and routine workers (by about 6 years). Table S1 presents socio-demographic data as well as median life expectancy ratios for the whole sample as well as separated by socio-demographic characteristics. Mean estimates of life expectancy (calculated by averaging across estimates for professionals and routine workers) were 5.55 years lower in the US than UK sample.

**Fig. 1:**
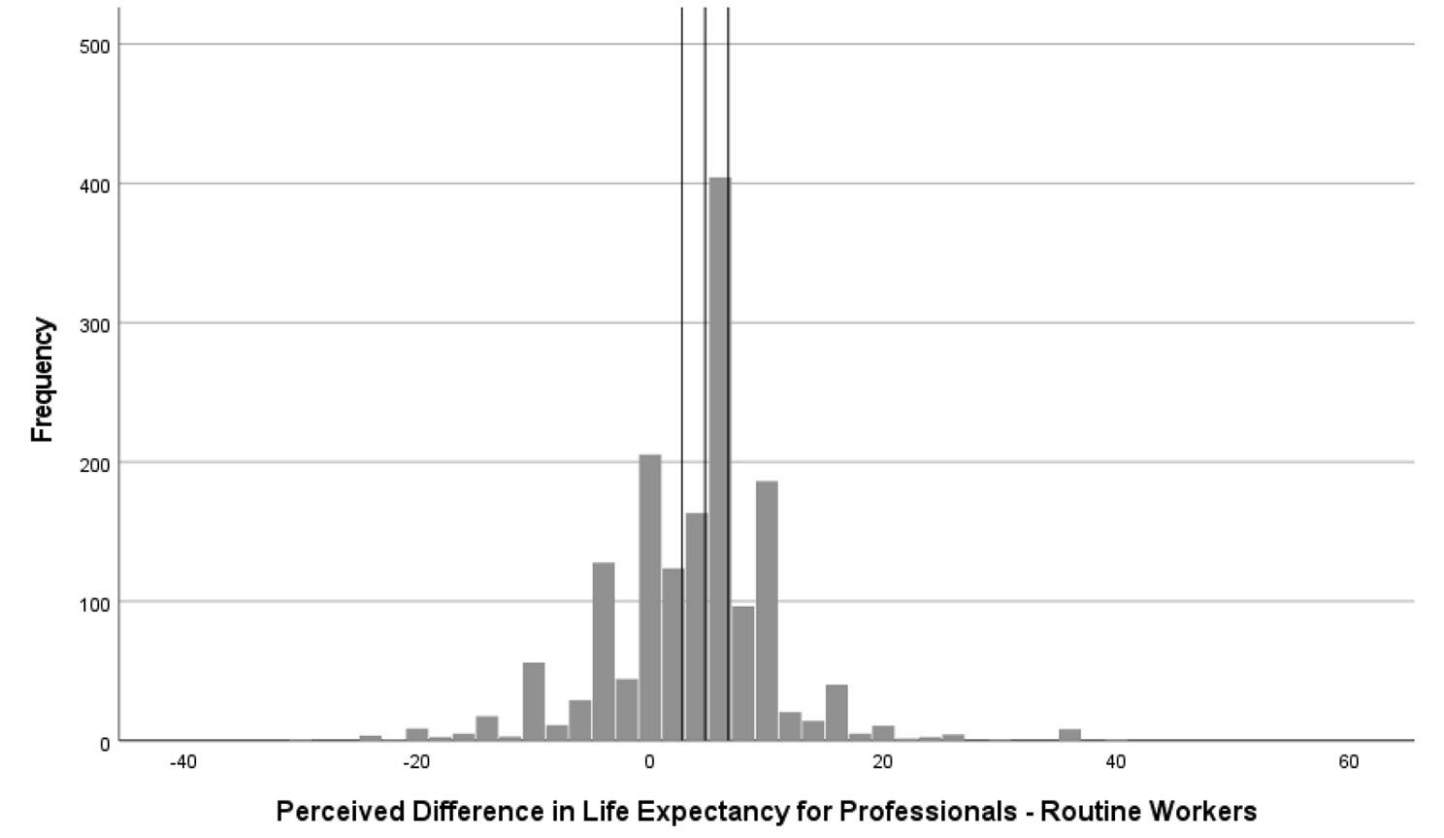
Distribution of perceived life expectancy difference responses around the actual difference in life expectancy between professional and routine workers (UK; N = 1,599). Lines intersecting the X axis represent (from left to right): actual difference – 2 years, actual difference (4.7 years), actual difference + 2 years

The difference in UK respondents’ estimates for Professionals and Routine workers was used as a measure of perceived magnitude of socioeconomic inequality in life expectancy. Differences ranged from − 29 to 30 and the modal difference in estimates (5 years; 21.7% of the sample gave this response) approximated the true value of 4.7 years. 35.5% of the sample gave responses within +/-2 years of the actual difference. The distribution of these responses around the actual difference is shown in Fig. 1.

The median estimated life expectancy ratio for the UK sample was 1.0588 (IQR = 0.0856) which is very close to the true value as reported in ONS age of death data from the 2007–2011 period (86.1/81.4 = 1.058; ONS, 2017). The median estimated life expectancy ratio for the US sample was 1.0291 (IQR = 0.1460). The median ideal lifespan ratio for the two countries was 1.0000 (UK IQR = 0.0256; US IQR = 0.0526), indicating a desire for full equality by the median respondent in the two samples. Estimated lifespan ratios were significantly higher than ideal life expectancy ratios in both the UK (*T* = 242,396, *z* = -16.072, *p* < .001) and the US (*T* = 124,211, *z* = -5.758, *p* < .001), revealing a consistent desire for reduced inequality in lifespan relative to the status quo as perceived by respondents themselves. Whilst there is variation in the median estimated lifespan ratios across demographic sub-groups, ideal life expectancy ratios remained at 1.0000 for all sub-groups and across both countries (see Table S1).

### Awareness of life expectancy differences by occupation

**Fig. 2:**
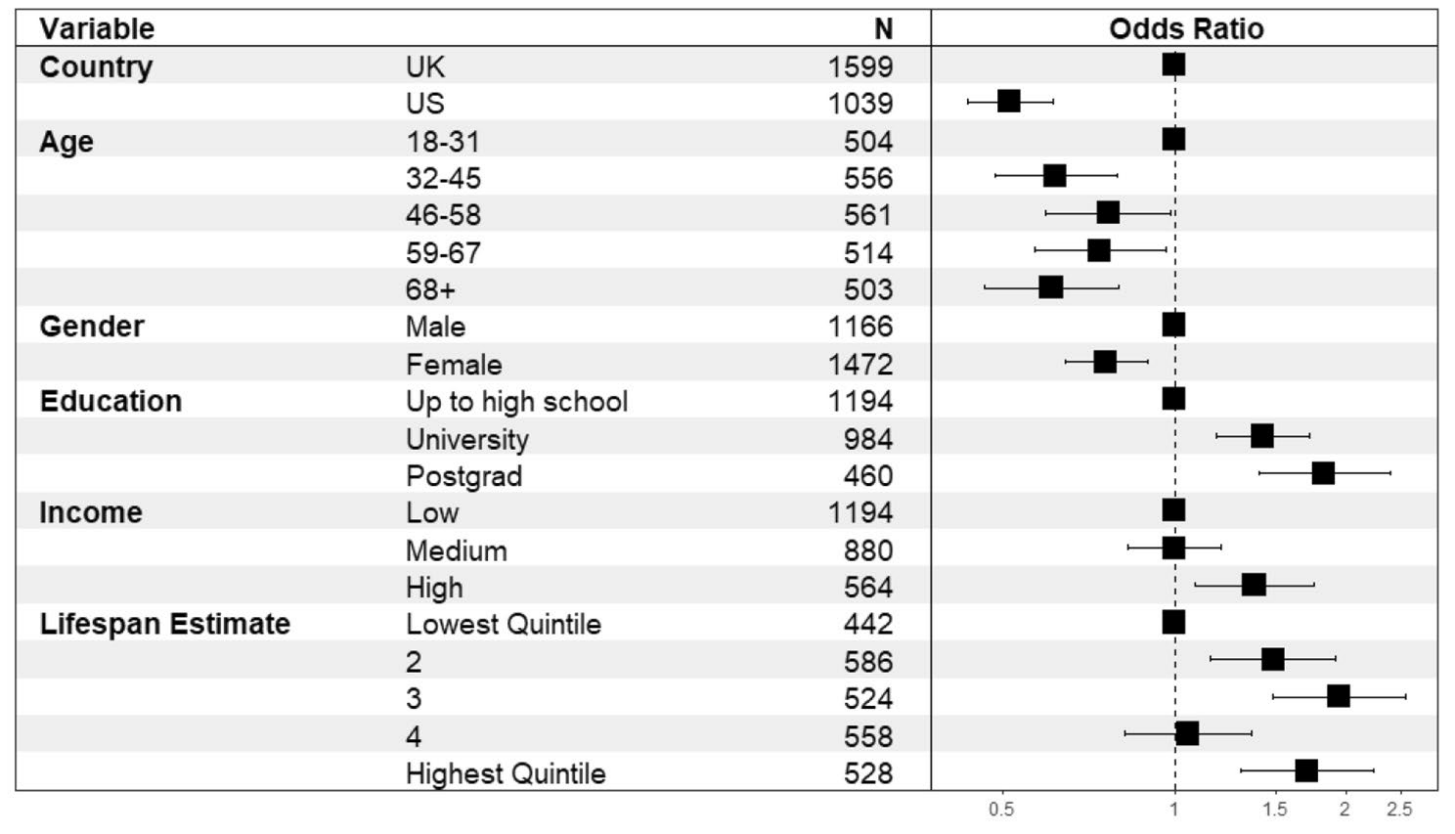
Forest plot showing odds ratios (and 95% confidence intervals) of perceiving higher life expectancy for professional than routine workers in the two samples (N = 2,638)

67.8% of the UK sample and 53.8% of the US sample correctly estimated that professionals have longer life expectancy than routine workers. A multivariate logistic regression including all variables available for both samples was conducted to determine which socio-demographic variables were associated with perceiving that life expectancy favours professionals relative to routine workers. These models also included adjustment for the size of participants’ lifespan estimates (averaged over the two categories and then categorized into quintiles from smallest to largest estimates) in order to disentangle overall lifespan estimates from estimates of *difference*. This is necessary to address possible scaling effects that may arise for example if participants who estimate smaller differences between groups do so because they generally perceive lifespans to be lower on average. Figure 2 depicts the outcome and corresponding odds ratios for this model and shows that university and postgraduate education (relative to high school education), being under 32 (relative to older respondents), higher income (relative to low income) and male (relative to female) were associated with greater likelihood of indicating professionals have longer lifespan than routine workers. This tendency also increased for those who indicated greater life expectancy overall. Respondents in the US sample were approximately half as likely as those in the UK to indicate life expectancy inequality. See supplemental figures for comparable outcomes for analyses without weights (Fig S1) and on non-imputed data (Fig S2).

**Fig. 3:**
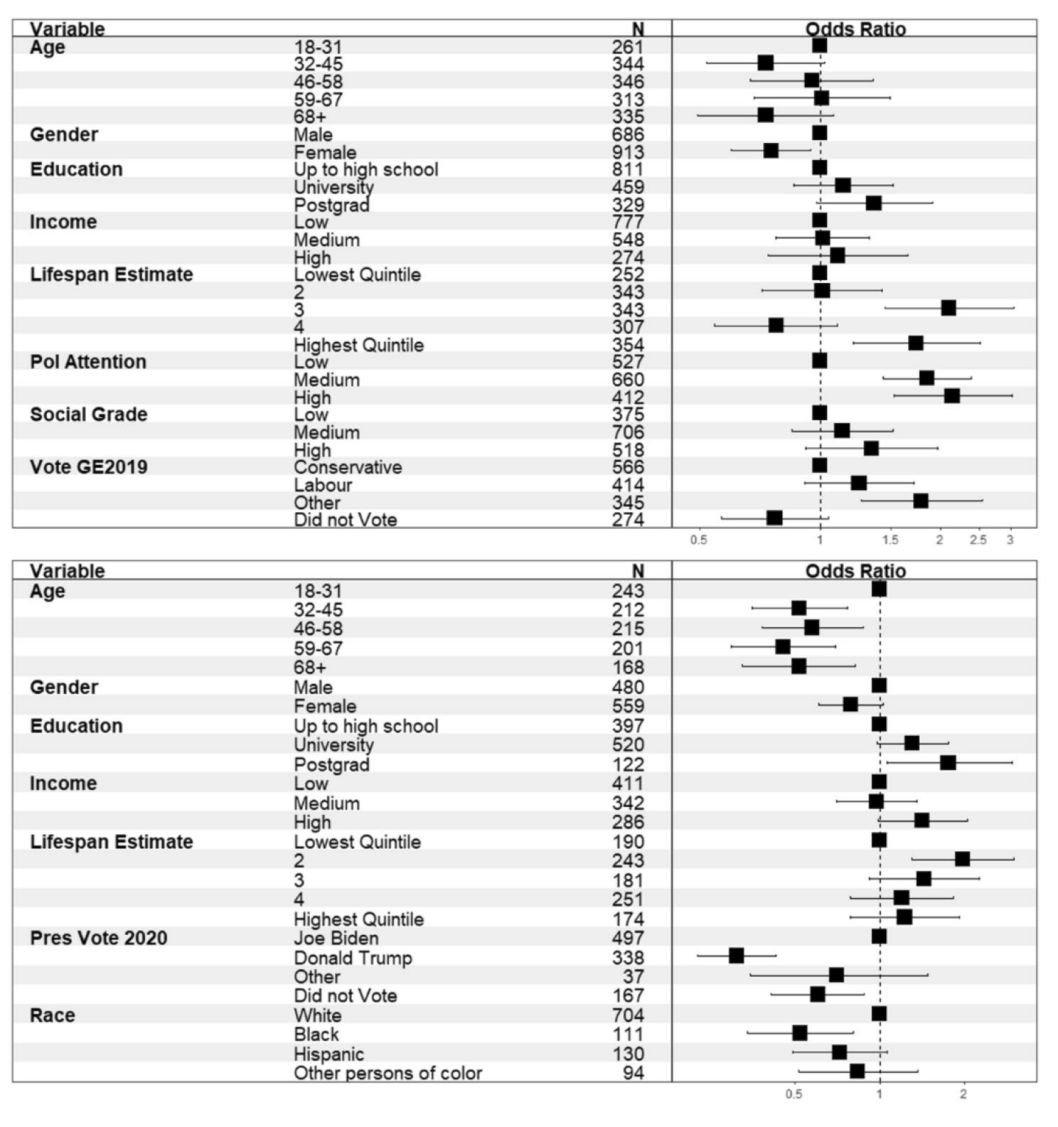
Forest plot showing odds ratios (and 95% confidence intervals) of perceiving higher life expectancy for professional than routine workers for the two samples (upper panel: UK = 1,599; lower panel: US = 1,039).

Follow-up country-specific analysis on the UK sample (N = 1,599), revealed that the effect of age was not significant in this sample and model (see Fig. 3). Likelihood of being aware of life expectancy differences was significantly higher for male respondents, those who made higher lifespan estimates overall, higher levels of political attention and voting for parties other than the main two (relative to Conservative) in the 2019 general election. There was no significant effect of social grade or income in this sample. In a comparable model for US respondents (N = 1,039), being over 31 was associated with lower awareness, as was voting Donald Trump relative to voting for Joe Biden in 2020, and identifying as Black relative to White.

**Fig. 4:**
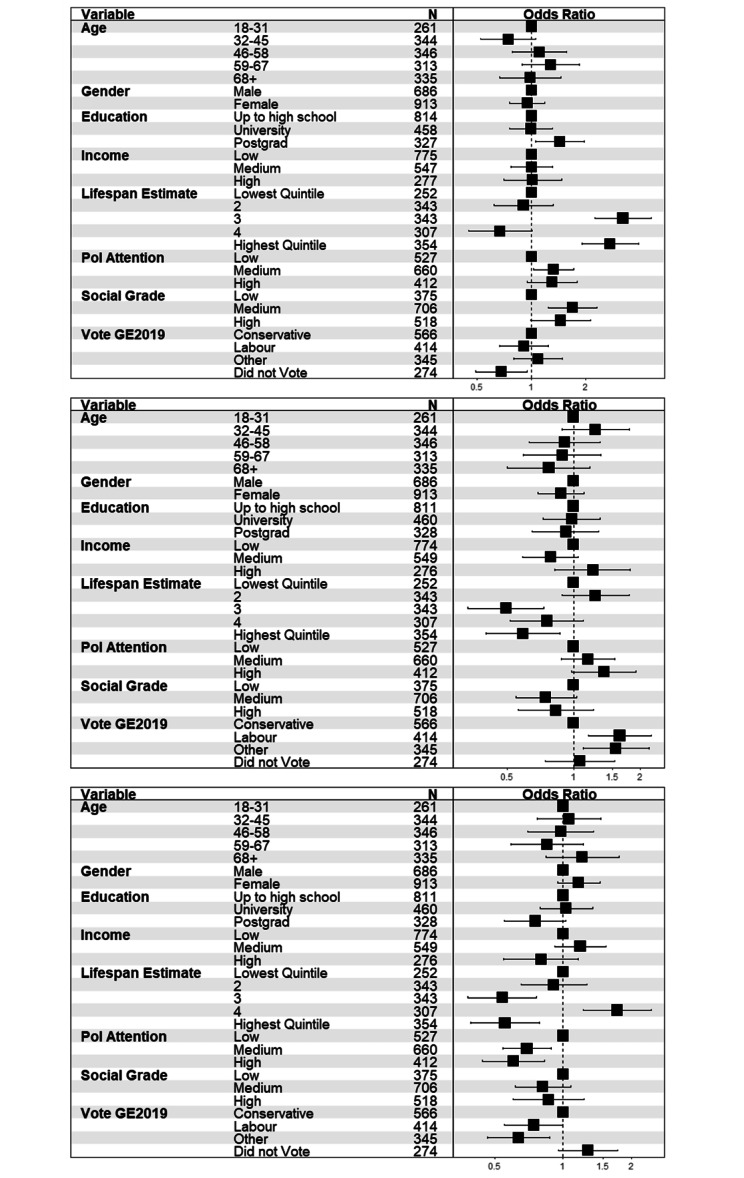
Forest plot showing odds ratios (and 95% confidence intervals) of having responses within 2+/- years of actual (upper panel), overestimates (middle panel) and underestimates (lower panel) of life expectancy differences between professional and routine workers in the UK sample (UK = 1,599).

To make best use of the correspondence between participants’ perceptions and actual data on lifespan for different NS-SEC groups, we also examined which respondent characteristics were associated with being within +/- 2 years of the correct difference in life expectancy between professional and routine workers (86.1–81.4 = 4.7), as well as overestimating differences (perceived gap > 6.7 years) and underestimating differences (perceived gap < 2.7 years). Figure 4 depicts that UK respondents with a postgraduate education, those with medium levels of political attention, medium or high social grade and those who generally made higher lifespan estimates on average were more likely to be within +/-2 years of the correct difference, whilst those who did not vote in the 2019 election were less likely to be within +/- 2 years. The principal characteristics that were associated with overestimating or underestimating differences was political attention and voting behaviours. Specifically, being a Labour or Other voter relative to Conservative voter was associated with higher likelihood of overestimating (and reduced likelihood of underestimating lifespan differences).

### Desiring equal life expectancy for professional and routine workers

**Fig. 5:**
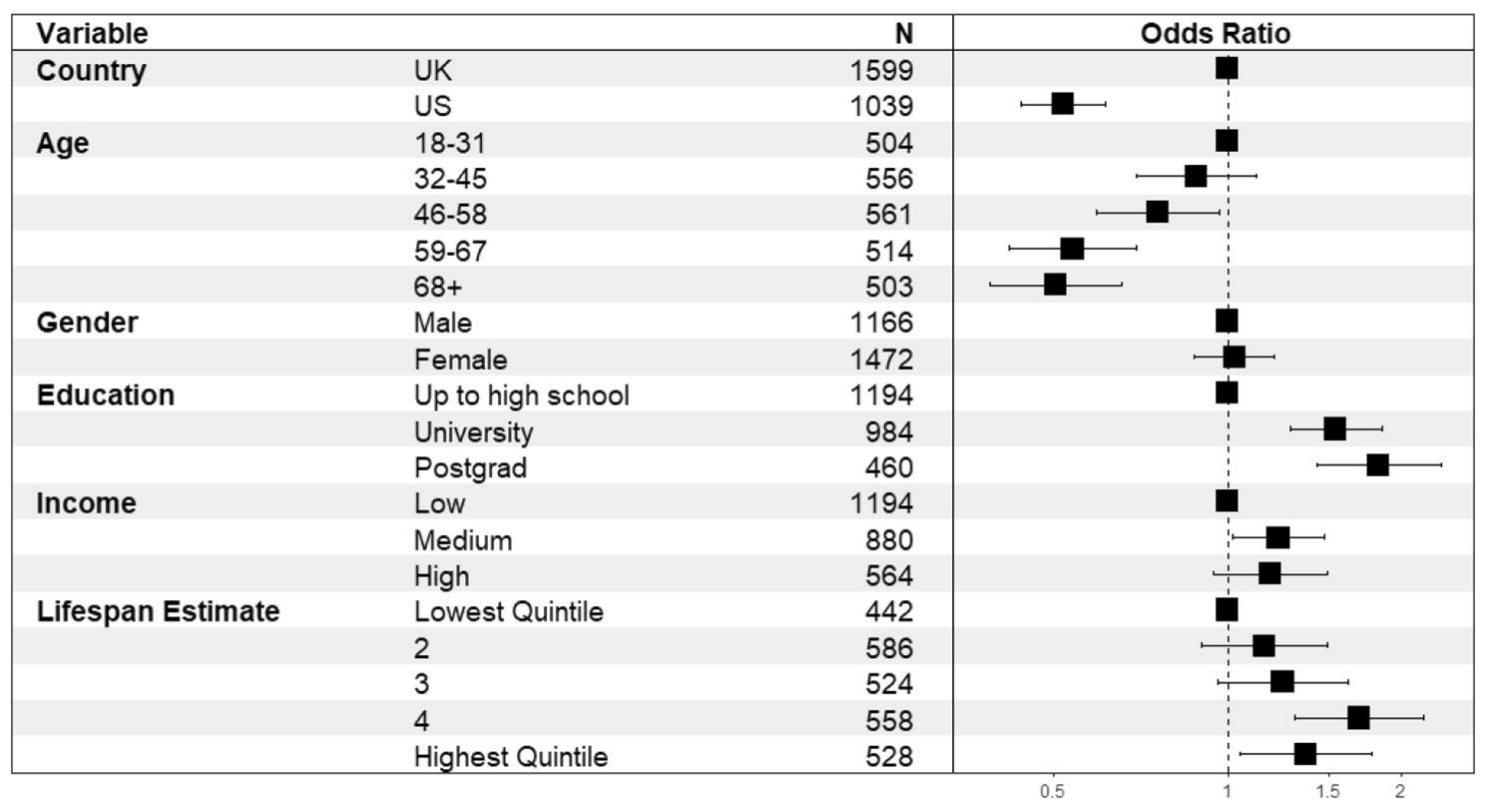
Forest plot showing odds ratios (and 95% confidence intervals) of desiring equal life expectancy for professional and routine workers in the two samples (N = 2,638)

60.1% of the UK sample indicated a desire for equal life expectancy for the two occupational groups. The proportion of US respondents indicating a desire for equal life expectancy was lower: only 46.7% of the US sample indicated the same life expectancy for the two groups. Figure 5 depicts the outcome of the cross-country multivariate logistic regression analyses to assess whether likelihood of desiring equal life expectancy varied with socio-demographic characteristics. There were no differences by gender in this analysis, but being under 45, having higher levels of education, medium (relative to low income) and estimating overall life expectancy to be high were independent predictors of indicating life expectancy should be the same for the two occupational groups. Respondents in the US sample were nearly half as likely as those in the UK sample to indicate that life expectancy should be the same for professionals and routine workers. Supplemental figures S3 and S4 show comparable analyses without weights and without income.

**Fig. 6:**
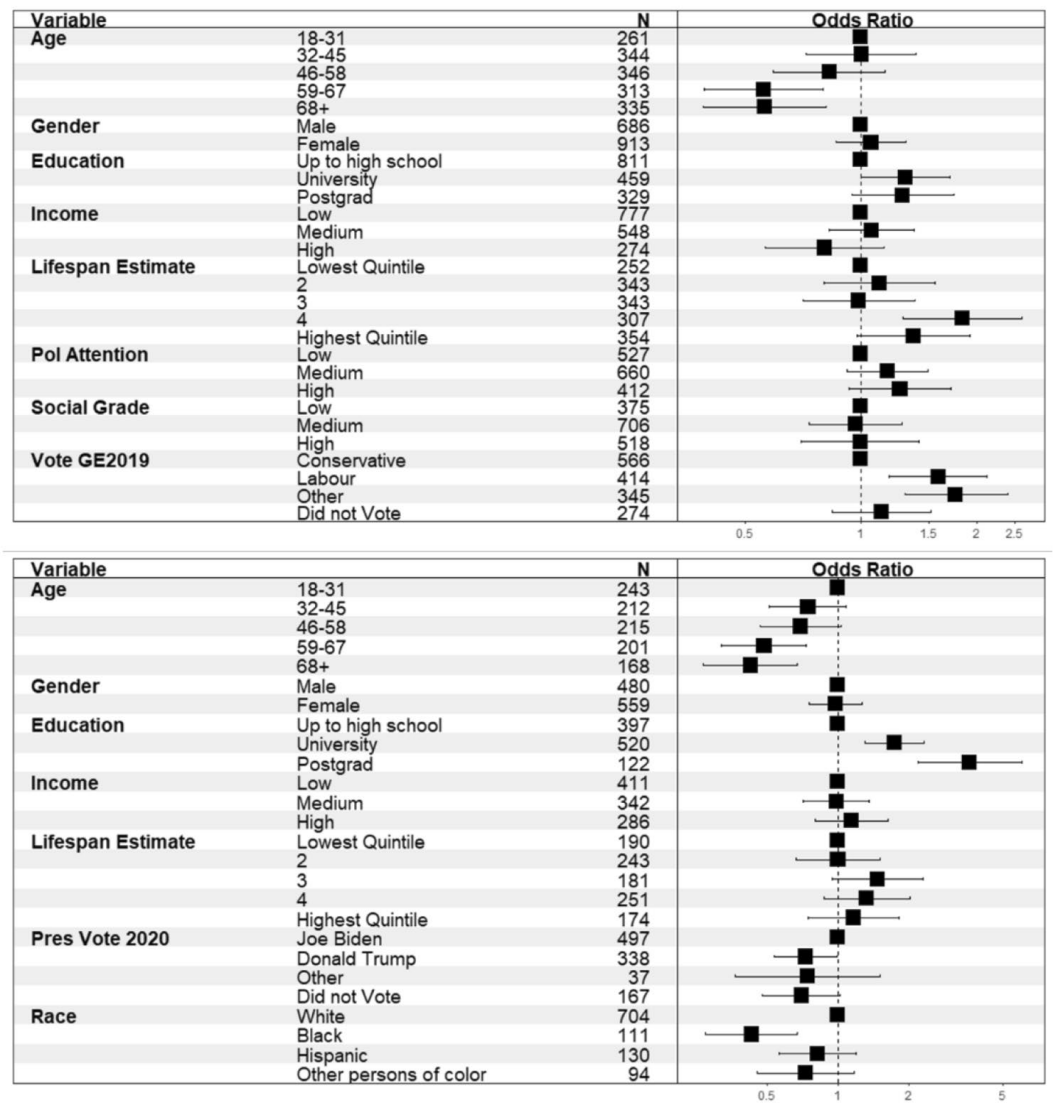
Forest plot showing odds ratios (and 95% confidence intervals) of desiring equal life expectancy for professional and routine workers for the two samples (upper panel: UK = 1,599; lower panel: US = 1,039).

In the UK (N = 1,599, see Fig. 6), desire for equality was more likely for Labour and Other party voters (relative to Conservative voters). Those who made greater life expectancy estimates were also more likely to indicate a desire for equality. Participants under 60 were significantly more likely to indicate a desire for equality in life expectancy relative to older participants. There were no effects of political attention, household income, social grade or gender.

Similar effects of age were evident in the US respondents (N = 1,039) and participants who were more highly educated were also more likely to indicate a desire for equal life expectancy. Participants who identified as Black (compared to White) and Trump voters (relative to Biden voters) were significantly less likely to indicate a desire for equality in life expectancy between professionals and routine workers. Desire for equal life expectancy did not vary with estimated life expectancy (see Fig. 5).

## General discussion

Perceived and ideal life expectancy inequalities between members of different occupational social class were measured using an intuitive and objective scale, years of life. In the UK, where there are objective data with which to compare and a longer history of studying health by occupational group, the median respondent captured actual lifespan differences between the two categories very well: they estimated the proportion of extra lifespan that professionals have relative to routine workers at 5.9% versus a true value of 5.8%. Over a third of the sample provided estimates within +/- 2 years of the correct difference. We find that the UK population is generally very aware of a life expectancy gradient that benefits Professional workers relative to those in “Routine” work.

There was some variation within the UK sample as to who was likely to perceive this kind of inequality. Awareness of occupational health inequality in the UK sample was associated with greater levels of political attention, education and certain voting preferences. It was reduced for female respondents and was more likely for those who estimated life expectancy to be greater in general. There was a trend for more awareness at higher social grades (which resembles but is not directly transposable to an individual’s occupational social class) but this was not significant. Whether awareness is socially graded itself is important in light of considerable evidence showing that individuals from relative disadvantage articulate how material and structural factors interact to determine health [58, 59] and are more likely to agree that poverty is a health determinant than those with relatively higher income or education levels [60, 61]. At the same time, other work has shown that awareness of socioeconomic health inequality increases with social advantage [62, 63]. These previous studies asked participants whether they agree that “the rich are much healthier than the poor,” which might invoke self-presentational concerns and conflate a moral imperative to health. Our approach to assessing perceptions of existing health inequality should not have suffered from this issue; at no point did we refer to “rich”, “poor”, or “health” or make evident our intent to directly compare responses for the two groups. From the respondent’s perspective, our survey simply asked how long groups of people live and how long they should live. Under these conditions, there was no strong evidence that awareness of occupational life expectancy inequalities in the UK varies with social grade.

We did, however, obtain evidence that respondents at medium and higher social grades (as well as postgraduate education) were more likely to estimate a lifespan gap that was within +/- 2 years of the actual gap. Whilst awareness did not appear socially graded, to some degree, precision of responses was. Examining the correspondence between perceived and actual inequality is important because it is known that perceptions of inequality predict support for redistributive policies [16] and that perceptions in turn are influenced by multiple socio-cognitive factors such as one’s comparison points [13] and ideology [52]. The current analyses also correspond with evidence that political liberals tend to overestimate income inequality [64], in that they show that Labour and other non-Conservative voters were more likely to overestimate existing lifespan differences between professionals and routine workers.

In contrast to the UK sample, respondents in the US sample were much less likely to perceive existing life expectancy inequality by these occupational groups. One possibility is that the socioeconomic gradient in lifespan by occupational groups is in fact smaller in the US than the UK. This seems unlikely. For the reasons recounted above relating to different historical focuses on occupational social class in the two countries [32], direct comparisons with the same occupational or social class measures are not possible. Work with other socioeconomic-related factors, however, shows that income gradients in health are estimated to be comparable in the two countries [65] or even steeper in the US than the UK [66]. One possibility is that, in line with sociocultural differences in the meaning of occupations in the two nations, as well as the corresponding paucity of data on health and life expectancy by occupational social class and the distinct academic public health attention on the domain of work in the States, the US-public in turn is less familiar with the notion that health outcomes differ by occupation, when occupational hazards or the term “social class” is not made salient [67]. One possible consequence of greater inclusion of the role of work within the study of social determinants of health in the United States, therefore, is greater public awareness of the relationship between working conditions and health.

We also find that the desire for absolute equality in lifespan for the two groups was significantly less common in the US sample, although the median and modal response for this sample was also one. A mechanism that might partially explain this result is that the US population is generally more tolerant of inequality than other developed countries [39]. This greater tolerance might in turn arise because of a greater tendency to believe that inequality arises as a consequence of fair and meritocratic processes, given meritocratic beliefs have been found to be comparatively higher in the United States than other western countries and to have risen with income inequality [68, 69]. The current data indicate that this inequality tolerance in the US may extend to health outcomes, at least when these are framed by occupation. Despite these differences between the two samples, however, we observe some consistencies across countries in that recent voting behaviours were associated with desires to eliminate lifespan inequality. This resonates with recent work showing the role of political orientation in explaining variance in support for intervention on health inequality, as well as the role of certain attributional styles that co-vary with political orientation [53]. Although political views thus appear to be influential in views of health inequality, as with other forms of inequality, it should be noted that across both samples, ideal lifespan inequality was significantly lower than perceived inequality and a plurality of respondents desired absolute socioeconomic equality in life expectancy.

60.1% of the current UK-sample indicated life expectancy differences should be eliminated. This is markedly higher than another representative sample of UK residents who were asked “What is an acceptable difference between how long the richest 5% and poorest 5% can expect to live?” [17] Only 46% of that sample answered “no gap” in life expectancy. Why might desires for equality be more prevalent in the current UK nationally representative sample and design? One explanation lies in the fact that whilst previous work in this area has typically captured perceptions of health across different income or wealth groups [17, 19, 49, 62, 63, 67], this is the first report of desires for health inequality relating to occupational groups. It has been shown that ratings of acceptability of health inequality vary according to the basis (e.g., neighbourhood, income, education, lifestyle choices or social class) to which they are attributed (e.g., differences in life expectancy are mainly due to differences in income) and that this arises in part because different framings elicit different causal models for health as well as views on how inevitable or malleable inequality is [18]. In other words, asking about life expectancy by occupation may elicit different appraisals about the reasons for health inequality and how much can be done about it in comparison to asking about life expectancy by wealthy extremes.

### Strengths and limitations

One strength of the novel approach employed here is the care taken in designing our questions to minimise self-presentational concerns and to avoid using procedures or question wordings that would convey our own value judgments on the question of health inequality or indicate that a certain level of inequality might or might not be acceptable. At no point in our questions did we mention concepts of “equity”, “equality”, “fairness” or “an acceptable gap”. We consider these results to be the cleanest manifestation yet of public desires regarding health equality across occupational groups. Transposing an approach employed in previous work on estimated and ideal pay ratios [45], they also permit contrasts not only with perceptions in that domain but also permit direct comparisons between estimates, ideals and (where available) actual lifespan inequality data. At the same time, however, they have not been psychometrically validated, are not easily transposed to forms of health inequality other than lifespan [70] and may be difficult for some participants to answer. Despite repeatedly piloting the questions with samples in both countries, there were differences in the proportion of each sample who provided responses that were interpretable as lifespan estimates. It remains possible that the question formulation was more difficult for US residents to interpret and that this may have influenced their estimates. Indeed, responses in the US sample were in general much more heterogeneous than for the UK. Any such bias should have impacted both estimates comparably and is unlikely to have influenced the final ratios reported here because we applied the same exclusion criteria to both samples and updated post-stratification weights accordingly to ensure the final samples remained nationally representative. Nonetheless, further research with these kinds of questions is required to determine how exactly participants respond to them. In the meantime, it is pertinent to note that this measure of estimated gap in lifespan does co-vary with health inequality aversion preferences (specifically, extreme egalitarianism and health maximising) derived from stated preference designs favoured by health economists, [50] providing reassurance that these responses correspond well with other approaches to assessing views on this topic. Accordingly, we view this approach as only one amongst an arsenal of techniques for calibrating public views on health inequality, albeit one that uniquely permits direct comparison of public views with real-world data.

We were reliant on YouGov’s existing socio-demographic measures, which were not equally available or comparably measured across the two countries. A measure of social grade was available for the UK population [54] and we find that whilst it was not related to awareness of inequality or desires for equality, it did differentiate those participants whose estimates were closest to the true estimate. Whilst this social grade measure does resemble the NS-Sect [21] it does not correspond directly with it and so it was not possible to determine whether perceptions of and desires for life expectancy gradients for professionals and routine workers varied according to whether participants themselves fell into these groupings. More problematic is the lack of any measure approximating occupational social class in the US sample removing the possibility of assessing this entirely. In this way, the current study is influenced by the same difficulties in obtaining relevant occupational class and health data beset by the wider US population health research community [32] and which further complicates cross-national comparisons of this kind.

## Conclusions

We presented a novel approach to measuring perceived and ideal life expectancy inequality by occupational groups. We consider these results to be the cleanest manifestation yet of UK- and US- perceptions of existing occupational-related life expectancy inequality as well as both UK- and US-public desires on this topic. The results reveal a widespread understanding of the social gradient in lifespan and a desire that social class inequalities in lifespan be eliminated in the UK, but considerably less awareness and concern in the US.

### Electronic supplementary material

Below is the link to the electronic supplementary material.

Supplementary Material 1

## Data Availability

The datasets used and/or analysed during the current study available from the corresponding author on reasonable request.

## Footnotes

1 “Class” is a complex construct with multiple meanings and interpretations, one of which is as an indicator of status or prestige and another as a construct that captures structures of material and social inequality (Crompton, 2008). Whereas the latter is often used in academic discourse, the former is understood to be closer to the lay definition and everyday use of the term. As we are precisely interested in lay interpretations of academic measures, we do not necessarily favour either interpretation here when using the term. We are, however, careful not to use the terms “class” or “social class” in any of our survey materials to minimise the impact of this and ensure we examine views of occupational groups themselves, rather than lay understandings of “class”.
