# Supplementary material for: Perceived health inequalities: are the UK and US public aware of occupation-related health inequality, and do they wish to see it reduced?
Source: BMC Public Health. 2023 Nov 24;23:2326. doi: 10.1186/s12889-023-17120-6 (PMC10668500; doi:10.1186/s12889-023-17120-6)

**Perceived health inequalities: Are the UK and US public aware of occupation-related health inequality, and do they wish to see it reduced?**

**Supplemental Materials**

**Supplemental Tables**

**Supplemental Table S1. Sociodemographic characteristics and median estimated and ideal life expectancy ratios of professional to routine workers**

|  | United Kingdom (*N* = 1,599) | | | United States (*N* = 1,039) | | |
| --- | --- | --- | --- | --- | --- | --- |
|  |  | Median Ratio Prof/Routine | |  | Median Ratio Prof/Routine | |
|  | *N* | Estimated | Ideal | *N* | Estimated | Ideal |
| **Total Sample (weighted)** | 1,599 | 1.0588 | 1.0000 | 1039 | 1.0291 | 1.0000 |
| **Age** |  |  |  |  |  |  |
| 18-31 | 320 | 1.0588 | 1.0000 | 264 | 1.0714 | 1.0000 |
| 32-45 | 371 | 1.0513 | 1.0000 | 231 | 1.0133 | 1.0000 |
| 46-58 | 351 | 1.0625 | 1.0000 | 200 | 1.0267 | 1.0000 |
| 59-67 | 280 | 1.0625 | 1.0000 | 187 | 1.0000 | 1.0000 |
| 68+ | 275 | 1.0482 | 1.0000 | 158 | 1.000 | 1.0000 |
| **Gender** |  |  |  |  |  |  |
| Female | 820 | 1.0533 | 1.0000 | 533 | 1.0256 | 1.0000 |
| Male | 779 | 1.0605 | 1.0000 | 506 | 1.0400 | 1.0000 |
| **Income** |  |  |  |  |  |  |
| <$40,000/£30,000 | 553 | 1.0625 | 1.0000 | 356 | 1.0259 | 1.0000 |
| $40,000-$80,000/£30,000 - £60,000 | 397 | 1.0532 | 1.0000 | 294 | 1.0258 | 1.0000 |
| >$80,000/>£60,000 | 188 | 1.0625 | 1.0000 | 234 | 1.0455 | 1.0000 |
| **Education** |  |  |  |  |  |  |
| Up to high school /Level 2-3 or equivalent | 828 | 1.0516 | 1.0000 | 419 | 1.0000 | 1.0000 |
| College / University | 423 | 1.0592 | 1.0000 | 511 | 1.0400 | 1.0000 |
| Post-grad | 284 | 1.0625 | 1.0000 | 109 | 1.0488 | 1.0000 |
| **Social Grade** |  |  |  |  |  |  |
| High | 336 | 1.0625 | 1.0000 | N/A | N/A | N/A |
| Medium | 614 | 1.0534 | 1.0000 | N/A | N/A | N/A |
| Low | 649 | 1.0493 | 1.0000 | N/A | N/A | N/A |
| **Political Attention** |  |  |  |  |  |  |
| High | 448 | 1.0626 | 1.0000 | N/A | N/A | N/A |
| Medium | 800 | 1.0625 | 1.0000 | N/A | N/A | N/A |
| Low | 352 | 1.0411 | 1.0000 | N/A | N/A | N/A |
| **General Election 2019 Vote** |  |  |  |  |  |  |
| Conservative | 524 | 1.0506 | 1.0000 | N/A | N/A | N/A |
| Labour | 387 | 1.0647 | 1.0000 | N/A | N/A | N/A |
| Other^a^ | 319 | 1.0629 | 1.0000 | N/A | N/A | N/A |
| Didn't Vote | 367 | 1.0286 | 1.0000 | N/A | N/A | N/A |
| **Race** |  |  |  |  |  |  |
| White | N/A | N/A | N/A | 664 | 1.0375 | 1.0000 |
| Black | N/A | N/A | N/A | 125 | 1.0000 | 1.0000 |
| Hispanic | N/A | N/A | N/A | 162 | 1.0351 | 1.0000 |
| Other persons of color^b^ | N/A | N/A | N/A | 88 | 1.038 | 1.0000 |
| **Presidential Election 2020 Vote** |  |  |  |  |  |  |
| Joe Biden | N/A | N/A | N/A | 485 | 1.0625 | 1.0000 |
| Donald Trump | N/A | N/A | N/A | 330 | 1.0000 | 1.0000 |
| Other^c^ | N/A | N/A | N/A | 38 | 1.0523 | 1.0000 |
| Didn't Vote | N/A | N/A | N/A | 186 | 1.0401 | 1.0000 |

^a^Liberal Democrat, SNP, Plaid Cymru, Brexit Party, Green, Other, Don’t Know

^b^Asian, Native American, Two or more races, Other, Middle Eastern

^c^Jo Jorgensen, Howie Hawkins, Other

**Figure S1.** Forest plot showing odds ratios (and 95% confidence intervals) of perceiving higher life expectancy for professional than routine workers for the two samples (N = 2,648), without sampling weights.


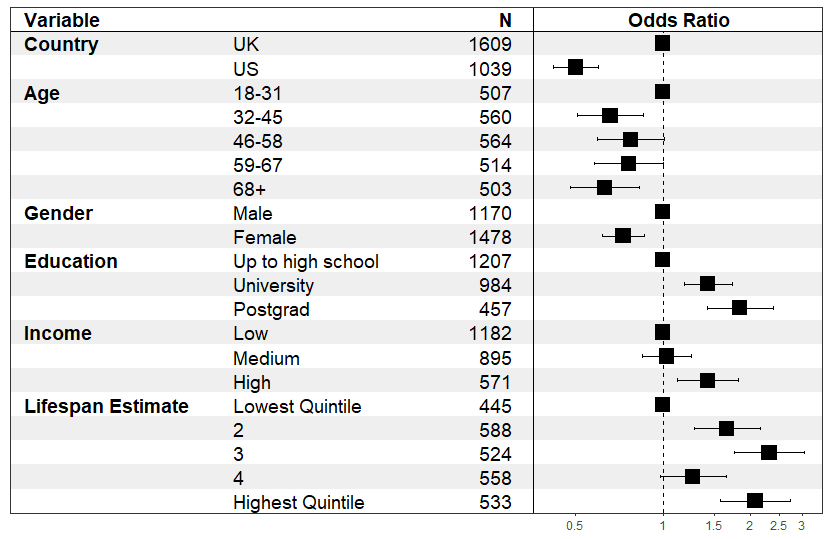


**Figure S2.** Forest plot showing odds ratios (and 95% confidence intervals) of perceiving higher life expectancy for professional than routine workers for the two samples (N = 2,584), on non-imputed data and without income. Sampling weights are applied.


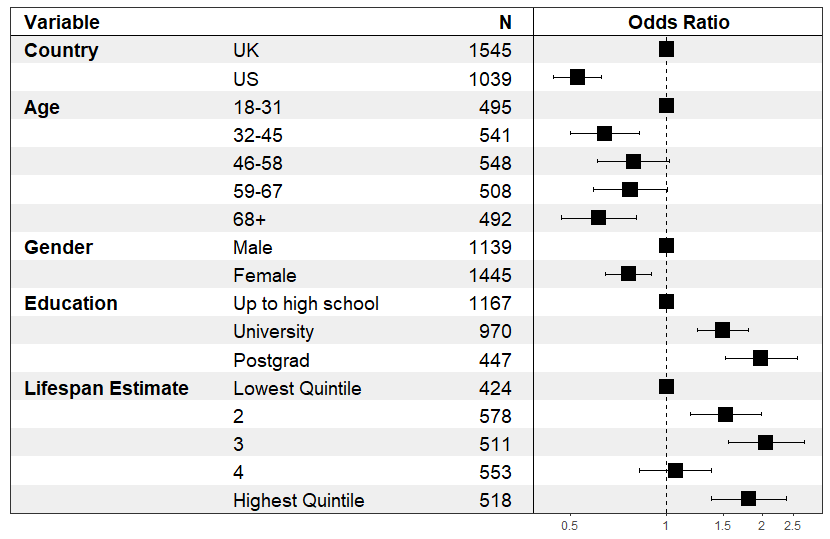


**Figure S3.** Forest plot showing odds ratios (and 95% confidence intervals) of desiring equal life expectancy for professional than routine workers for the two samples (N = 2,648), without sampling weights.


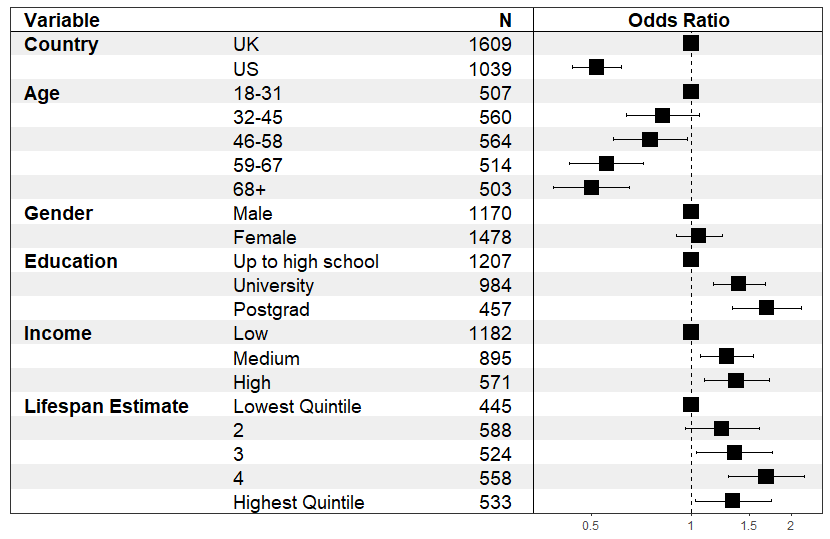


**Figure S4.** Forest plot showing odds ratios (and 95% confidence intervals) of desiring equal life expectancy for professional than routine workers for the two samples (N = 2,584), on non-imputed data and without income. Sampling weights are applied.


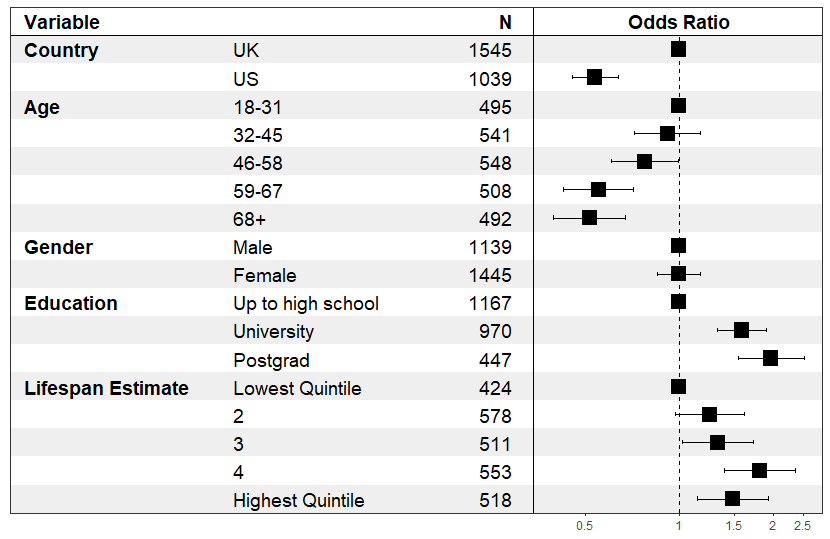

Supplement: Supplementary file 1 — Supplementary Material 1 [file 12889_2023_17120_MOESM1_ESM.docx]
